# Assessment of Anthropometric Indices for Optimal Cut-Offs for Obesity Screening in a South African Adolescent Population

**DOI:** 10.3390/biology10111118

**Published:** 2021-10-29

**Authors:** Godwill Azeh Engwa, Karin Schmid-Zalaudek, Chungag Anye, Boitumelo P. Letswalo, Paul Chungag Anye, Muhau Muhulo Mungamba, Constance Rufaro Sewani-Rusike, Nandu Goswami, Benedicta Ngwenchi Nkeh-Chungag

**Affiliations:** 1 Department of Biological and Environmental Sciences, Faculty of Natural Sciences, Walter Sisulu University, Mthatha 5117, South Africa; gengwa@wsu.ac.za; 2 Physiology Division, Otto Loewi Research Center for Vascular Biology, Immunology and Inflammation, Medical University of Graz, Neue Stiftingtalstrasse 6, D-5 A 8036 Graz, Austria; karin.schmid@medunigraz.at; 3 Dayenuel Consulting, Postnet Suites 092, Mthatha 5099, South Africa; foyarrh52@gmail.com; 4 Department of Human Biology, Faculty of Natural Sciences, Walter Sisulu University, Mthatha 5177, South Africa; bletswalo@wsu.ac.za (B.P.L.); mmungamba@wsu.ac.za (M.M.M.); crusike@wsu.ac.za (C.R.S.-R.); 5 MBCHB Programme, Faculty of Health Sciences, Walter Sisulu University PBX1, Mthatha 5117, South Africa; 214023362@wsu.ac.za

**Keywords:** obesity, anthropometric measures, cut-off values, South Africans of African ancestry

## Abstract

**Simple Summary:**

The diagnosis of obesity in sub-Saharan African children relies on cut-off values for body mass index percentile (pBMI) and waist-to-height ratio (WtHR) established in western populations. Hence, this study assessed anthropometric indices to determine optimal cut-off values for obesity screening in the South African adolescent population. Findings from this study showed that the cut-off value for pBMI was p85.2th, which improved the sensitivity of the test by approximately 30% compared to the CDC recommended BMI percentile of p95.0th. Moreover, the optimal cut-off for WHtR was 0.481, which was close to the recommended cut-off value of 0.5. This study reveals a lower pBMI cut-off value, different from the CDC recommended cut-off, for screening obesity in a South African adolescent population and suggests that the optimal pBMI cut-off for obesity screening may be ethnic-specific.

**Abstract:**

The assessment of obesity in sub-Saharan Africa relies on cut-offs established from western populations. This study assessed anthropometric indices to determine optimal cut-off values for obesity screening in the South African adolescent population. A cross-sectional study involving 1144 (796 females and 348 males) adolescents aged 11–17 years from the Eastern Cape Province of South African was conducted. Anthropometric parameters were measured. Receiver operating characteristic (ROC) analysis was performed to assess the sensitivity and specificity of obesity screening tools and establish cut-offs. The optimal cut-offs for obesity in the cohort using waist-to-height ratio (WHtR) as reference were: neck circumference (NC) = 30.6 cm, mid-upper arm circumference (MUAC) = 25.9 cm, waist circumference (WC) = 75.1 cm, hip circumference (HC) = 92.15 cm and body mass index percentile (pBMI) = p85.2th. The new pBMI cut-off value at p85.2th improved the sensitivity of the test by approximately 30% compared to the CDC recommended BMI percentile (pBMIr) of p95.0th. When pBMI was used as reference, the optimal cut-offs in the cohort were: WHtR = 0.481, NC = 30.95 cm, MUAC = 27.95 cm, WC = 76.1 cm and HC = 95.75 cm. The WHtR optimal cut-off of 0.481 was close to the recommended cut-off value of 0.5. The predicted prevalence of obesity obtained using cut-offs from ROC analysis was higher than those from recommended references. All cut-off values for the various anthropometric measures generally increased with age for all percentile ranges. This study reveals a lower pBMI cut-off value, different from the CDC recommended cut-off, for screening obesity in a South African adolescent population. The study has established that the optimal pBMI cut-off for obesity screening may be ethnic-specific.

## 1. Introduction

Childhood obesity is increasingly becoming a public health problem, as its prevalence is rapidly on the rise worldwide [1]. Recent reports estimate that over 40 million children below 5 years of age and over 330 million children and adolescents aged 5–19 years were overweight or obese in 2016 [2]. It has also been reported that children with obesity are more susceptible to metabolic and cardiovascular risk factors [3,4,5,6], such as dyslipidaemia [7], high blood pressure [5,8,9] and hyperglycaemia [10]. Several studies have indicated that these conditions may track into adulthood, leading to the development of chronic diseases linked with lifestyle changes such as diabetes, hypertension and cardiovascular diseases (CVDs) [11,12,13] associated with early morbidity and premature death [14].

Several anthropometric measurements, ratios and indices, including waist circumference (WC), waist-to-hip ratio (WHR) and body mass index (BMI), have been used to assess obesity and the risk of obesity-related diseases in children [15]. While WC and WHR have been considered as measures for abdominal or central obesity, BMI has been considered as a measure for general obesity. Waist-to-height ratio (WHtR) [16] and neck circumference (NC) [17], as well as mid-upper arm circumference (MUAC) [18], have also been suggested as being useful assessment tools for childhood obesity. Age- and sex-specific BMI percentile curves (pBMI) are the most frequently used measures for obesity screening in children of all ages, while WC and WHtR are specific for assessing abdominal fat in children [19,20]. A few studies claim that WHtR is not age or gender (sex) dependent and is therefore a better predictor for obesity-related cardiometabolic risk factors than WC or BMI in adults [21,22]. This corroborates the fact that central obesity poses a greater health risk than general obesity. However, in children, it remains unclear whether WHtR is better than WC or BMI in assessing obesity [23].

Researchers have tried to find simpler and easier strategies to distinguish between children with and without obesity. Based on research findings, the World Health Organization (WHO) [24], the United States Centers for Disease Control and Prevention (CDC) [25] and the International Obesity Task Force (IOTF) [26] have established cut-offs for obesity screening using pBMI. While the WHO pBMI cut-off relies on data from only healthy children, the IOTF cut-off is complex to extrapolate, as it links the cut-off for children to the BMI of 18-year-old adults at 25 and 30 kg/m^2^. As such, the CDC cut-off is preferable for the children population because it has no restrictions on their health status. According to the CDC, pBMI higher than the 95th percentile (≥p95) is indicative of obesity in children [25]. Ashwell et al. (2012), on the other hand, used the ratio of WC to height (WHtR) to determine a cut-off value of 0.5, which is widely accepted for indicating obesity in both children and adults, irrespective of sex [27]. However, these cut-off points (pBMI and WHtR) were derived from Caucasian populations and are also recommended for assessing obesity and cardiometabolic risks in people of African ancestry, without consideration for African growth patterns [28]. Although the birth to 22 years cohort study in South Africa showed association between relative weight gain and abdominal adiposity outcomes concurring with findings from western populations, it did not show a relative linear growth relationship with adiposity in the adolescent age group [29]. There is evidence that ethnic diversity could be accountable for differences in measures of adiposity and cardiometabolic risk, as several studies have shown varying cut-offs for obesity in different populations [19,30].

The prevalence of obesity in South Africa is steadily on the rise [31], especially in the paediatric population [32]. Recent reports show an increase in the prevalence of obesity in South African children [33] and adolescents [34] of African ancestry, using cut-off points established from western populations. To date, no cut-off values have been established for obesity screening in the South African adolescent population. This study aims to assess anthropometric indices to determine optimal cut-off values for obesity screening in a South African adolescent population.

## 2. Materials and Methods

### 2.1. Study Population and Design

This was a cross-sectional study that included 1144 (348 males and 796 females) South African adolescents aged 11–17 years old recruited from Libode, Mthatha, East London and Alice of the Eastern Cape Province of South Africa from June 2016 to December 2017. The Eastern Cape Province is predominately dominated by South Africans of African ancestry.

### 2.2. Ethical Consideration

Ethical clearance was obtained from the Health Sciences Ethics Committee of Walter Sisulu University (Ref No: 112/2018) and University of Fort Hare (CH1011SCHU01), South Africa. After careful explanation of the aim and objectives of the study, written informed consent was obtained from the parents/legal guardians of the children before enrolment into the study. The study adhered to the standards of reporting and was in accordance with the South Africa National Data Protection Act and the identities of the participants were kept confidential.

### 2.3. Anthropometric Measurements

Anthropometric measurements were performed in accordance with the International Standards for Anthropometric Assessments [35] for all participants. Height was measured using a wall-mounted Harpenden stadiometer and recorded to the nearest 0.1 cm. Weight was measured using a Tanita weight scale (BC1000, Tanita Corporation, Tokyo, Japan) and body mass index (BMI) determined as weight/height^2^ (kg/m^2^). Obtained BMI was converted to BMI percentiles (pBMI) for age, sex and height as underweight: <5th percentile, normal weight: ≥5th to <85th percentile, overweight: ≥ 85th to <95th percentile and obese: ≥95th percentile [36]. The waist circumference (WC), hip circumference (HC), mid-upper arm circumference (MUAC) and neck circumference (NC) were measured in centimetres (cm) using an anthropometric tape. The waist-to-height ratio (WHtR) was calculated from WC and height, and a cut-off value of 0.5 was used to classify obesity, as previously reported [21].

### 2.4. Data and Statistical Analysis

The prevalence (%) of obesity was calculated as (presence of obesity/total population) ×100. The specificity and sensitivity of obesity by pBMI, using WHtR as reference, were calculated from true-positive (TP), true-negative (TN), false-negative (FN) and false-positive (FP) results. Sensitivity was calculated as TP/(TP + FN) × 100 and specificity as TN/(TN + FP) × 100.

Data were analysed using Statistical Package for Social Sciences (SPSS) software (version 20, IBM SPSS Inc., 2011, Chicago, IL, USA). Mean differences between groups were analysed by t-test or ANOVA/ANCOVA, controlling for age and sex. Comparison of categorical variables between groups was performed using chi-square test of association. Receiver operating characteristic (ROC) curve analysis, as previously reported [37], was used as a screening tool to determine cut-off points for pBMI, WC, WHtR, HC, NC and MUAC to classify obesity. Sensitivity and specificity were determined from the ROC curve to evaluate the accuracy of the pBMI, WC, WHtR, HC, MUAC and NC classifications in the determination of obesity, using either pBMI or WHtR as reference. The ROC curve is a plot of the sensitivity (true-positive rate) against 1-specificity (false-positive rate) for each anthropometric measure. The area under the curve (AUC) is an indicator of how precise an anthropometric measure distinguishes a positive outcome. The AUC values range between 0 and 1. A value of 1 indicates an ideal performance while 0.5, indicated by a diagonal line, demonstrates that the anthropometric measure has no predictive performance. The Youden index (value of the largest sum of sensitivity and specificity −1) was used to determine the optimal cut-off value for each anthropometric index (BMI, WC, WHtR, HC, WC and MUAC). The data for all age groups were classified into percentiles for males and females, and percentile values identified. A difference was considered as significant at *p* ≤ 0.05.

## 3. Results

The characteristics of the participants are summarised in Table 1. Since females were on average slightly older (t_(1142)_ = 6.79, *p* < 0.001), age was considered as a covariate in the comparison of anthropometric variables by ANCOVA. Females were on average taller (F_(1, 1141)_ = 9.11, *p* = 0.003), but not heavier than males (F_(1, 1141)_ = 3.10, *p* = 0.079). Females had higher BMI and pBMI (F_(1, 1141)_ = 13.96, *p* < 0.001), WHtR (F_(1, 1141)_ = 6.61, *p* = 0.010), MUAC (F_(1, 1141)_ = 10.50, *p* = 0.001) and HCs (F_(1, 1141)_ = 14.42, *p* < 0.001) when controlling for age. No significant differences were found in NC (F_(1, 1141)_ = 0.90, *p* = 0.342) and WC (F_(1, 1141)_ = 2.46, *p* = 0.117) between males and females.

The prevalence of obesity based on pBMI was 15.5% (16.7% of females and 12.6% of males). There was no difference in the prevalence of obesity between females and males (χ^2^ = 3.06, *p* = 0.80). Based on WHtR, 25.3% of children (27.3% of females and 20.7% of males) had obesity. According to WHtR, obesity was more frequent in female than in male adolescents (χ^2^ = 5.54, *p* = 0.019, Table 2). As shown in Table 3, the prevalence of obesity was highest in the group of 16-year-old adolescents (24.6%) and lowest (10.7%) in 12-year-old adolescents. The prevalence of overweight status was 16.3% and was higher in females (17.3%) than in males (13.8%). Moreover, overweight status was highest in 15-year-old children (23.5%) and lowest in 12-year-old children (11.2%, Table 3).

The sensitivity and specificity of pBMI using WHtR as reference were assessed. Regardless of sex, pBMI presented a low sensitivity (54.0%), with a high specificity (97.5%) in the cohort. pBMI was more sensitive in identifying obesity in males (56.97%) than in females (53.0%) and more specific in identifying males without obesity (98.9%) than females (96.9%, Table 4).

**Figure 1:**
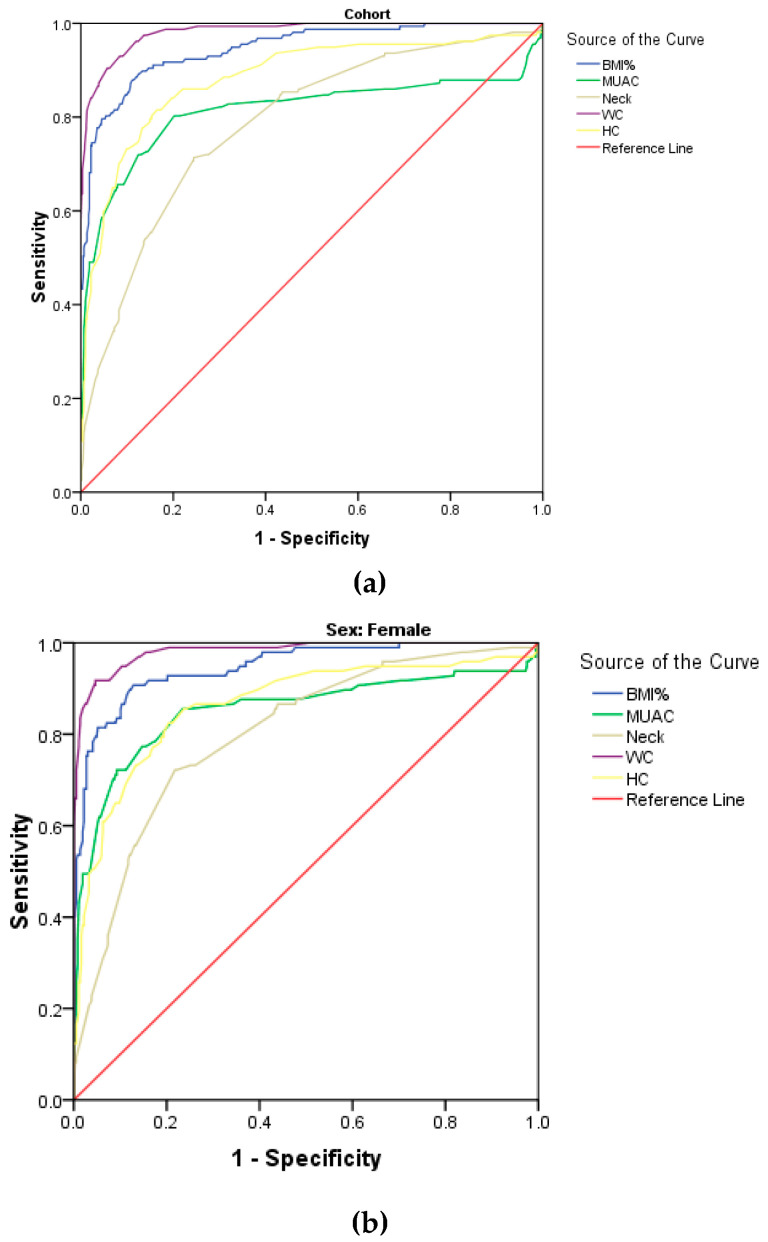

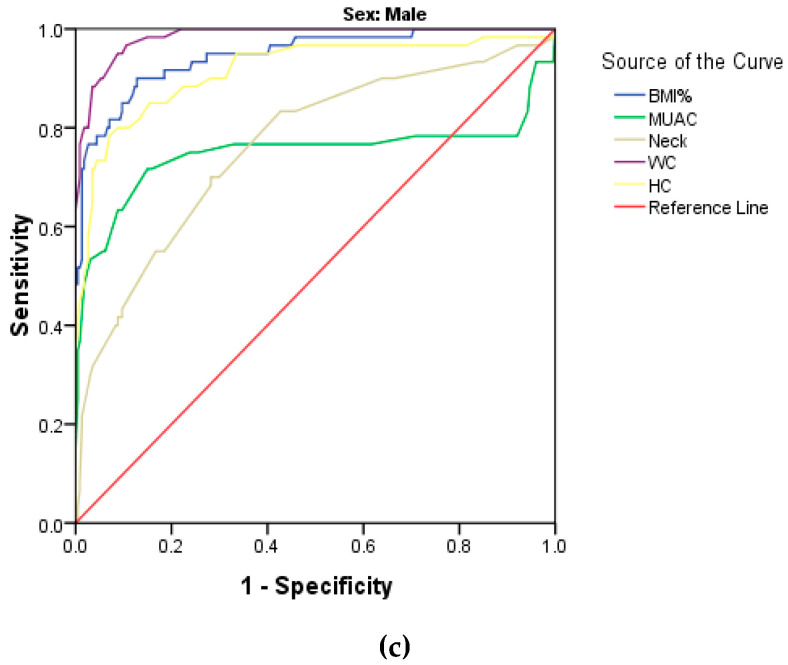
Receiver operating characteristic (ROC) curves for anthropometric measures using WHtR as reference in the cohort, females and males (**a**–**c**).

The ROC curve for pBMI, WC, HC, NC and MUAC using WHtR as reference, illustrated in Figure 1, showed that WC was the best indicator for obesity, having the largest AUC with high sensitivity and specificity in both males and females and in the cohort. This was followed by pBMI and HC, while NC was the poorest indicator (with the smallest AUC) in the cohort (Figure 1a), as well as in females and males separately (Figure 1b,c, respectively). The cut-offs, sensitivity, specificity and AUC for all anthropometric parameters using WHtR as reference are summarised in Table 5. The optimal cut-offs for obesity by NC, MUAC, WC, HC and pBMI in the cohort were 30.6 cm, 25.9 cm, 75.1 cm, 92.15 cm and p85.2^th^, respectively, when WHtR was used as reference. The cut-offs for WC and pBMI were slightly higher in females than in males, while the cut-off for NC, MUAC and HC was higher in males than in females. The obtained pBMI cut-off at p95th percentile showed a smaller sensitivity and higher specificity compared to the pBMI at p85.2th percentile in the study.

**Figure 2:**
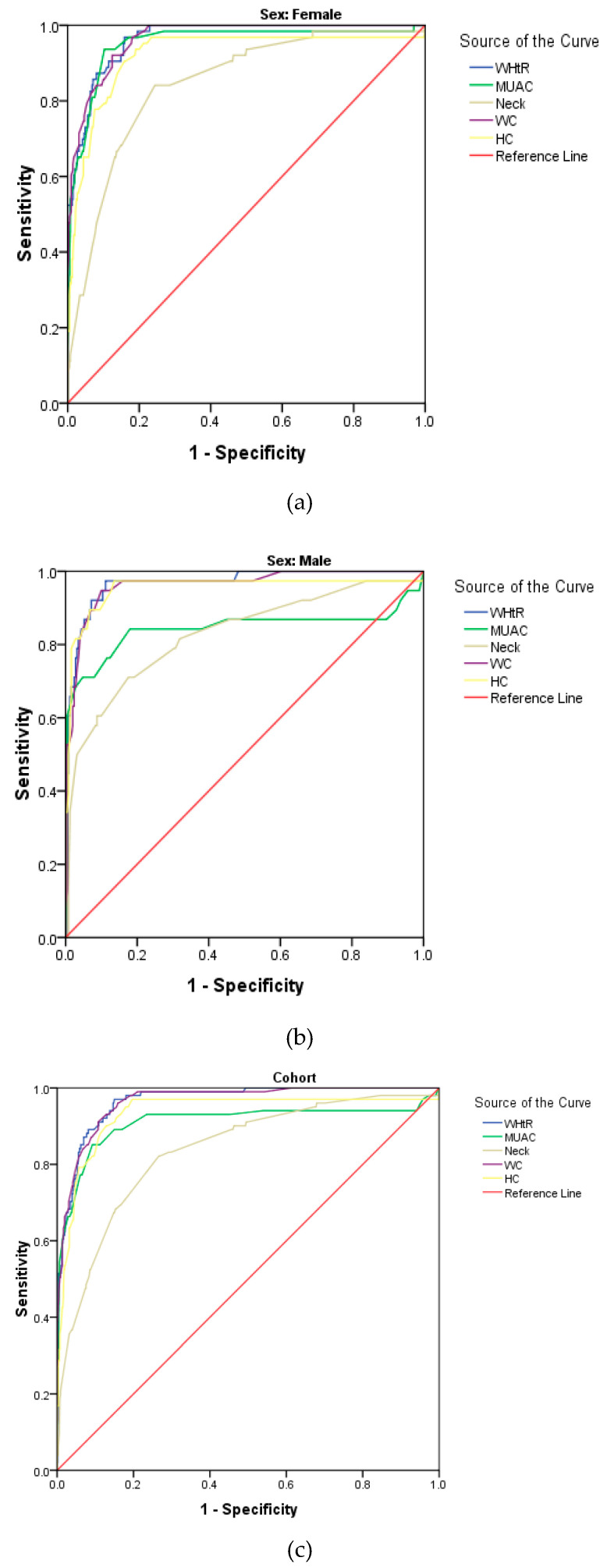
Receiver operating characteristic (ROC) curves for anthropometric measures using pBMI as reference in the cohort, females and males (**a**–**c**).

Figure 2 shows the ROC curve for WHtR, WC, HC, NC and MUAC using pBMI as reference, while the cut-off, sensitivity, specificity and AUC for all the anthropometric parameters are summarised in Table 6. The results showed that WHtR was the best indicator for obesity, having the largest AUC and highest sensitivity in the cohort (Figure 2a) as well as in females (Figure 2b) and males (Figure 2c), separately. This was followed by WC and HC, then MUAC in the cohort as well as in females and males. NC was the poorest indicator (with the smallest AUC) in the cohort as well as in males and females (Figure 2a–c).

Using pBMI as reference, the optimal cut-offs for obesity for WHtR, NC, MUAC, WC and HC in the cohort were 0.481, 30.95 cm, 27.95 cm, 76.1 cm and 95.75 cm, respectively. A similar trend was observed in males and females, except that the cut-offs for WHtR, NC, MUAC, WC and HC were slightly higher in females than in males.

The predicted prevalence of obesity, based on the new cut-offs established from the ROC analysis, is summarised in Table 7. The predicted prevalence of obesity obtained using cut-offs of the ROC analysis was higher than that of the observed prevalence of obesity in the study. Using WHtR as reference, the predicted prevalence of obesity for NC, MUAC, WC, HC and pBMI ranged from 21.8% to 38.8% in the cohort, 19.5% to 37.2% in females and 22.1% to 31.0% in males. The closest predicted prevalence of obesity comparable to that of WHtR (observed prevalence) was by NC (23.0%) in the cohort, WC (31.4%) in females and MUAC (22.1%) in males. When pBMI was used as reference, the predicted prevalence of obesity ranged from 12.9% to 32.1% in the cohort, 12.7% to 36.4% in females and 20.7% to 23.3% in males. The closest predicted prevalence of obesity comparable to that of pBMI (observed prevalence) was by MUAC (12.9%) in the cohort, NC (19.2%) in females and NC (20.7%) in males. The predicted prevalence using WHtR as reference was higher than that of pBMI as reference.

The various anthropometric indices were classified into percentile categories according to age and are summarised in Table 8. All percentiles of the various anthropometric measures (WHtR, pBMI, WC and HC) generally increased with age for all the percentile ranges. At the 95th percentile, WHtR ranged from 0.57 to 0.61 in females and from 0.52 to 0.64 in males. Values of the 95th percentile of WHtR were generally higher than the recommended cut-off of 0.5. It was at the 85th percentile in female children aged 11 years and males between 12 and 13 years that had values close to the recommended cut-off of 0.5. For pBMI, the 95th percentile ranged from p95th to p98.14th in females and from p94.0th to p99.07th in males, and the values increased with age. Most of the percentile values for all ages were above the recommended BMI percentile at p95th, which indicates childhood obesity. Moreover, it was the 85th percentile that tallied with the recommended BMI percentile of p95th, especially in females aged 13 to 17 years and in 11- and 14-year-old males. The 95th percentile for WC ranged from 89.6 cm to 99.7 cm in females and 92.50 cm to 102.68 cm in males. The 95th percentile values for HC ranged from 106.7 cm to 124.28 cm in females and from 105.75 cm to 115.95 cm in males.

## 4. Discussion

The increasing prevalence of obesity in South African children [38] presents a major public health concern, with the odds of increased CVD risk in adulthood [39]. Assessment of obesity in South African children and adolescents relies on the WHO [24], CDC [25] or IOTF [26] recommended cut-off values for BMI percentile, as well as the accepted cut-off for WHtR [27], mainly derived from Caucasian populations, which did not take into consideration genetic, ethnic and growth pattern differences existing in different populations. It was therefore necessary to assess anthropometric measures for screening obesity in order to establish cut-off values specific to this South African adolescent population. Applying the recommended pBMI(r) cut-off score at 95^th^ percentile and WHtR at 0.5, the prevalence of obesity was 15.5% and 25.3%, respectively. We used WHtR as the reference method for assessing the effectiveness of pBMI to screen obesity, as WHtR has previously been reported to be a better indicator than pBMI in assessing obesity in children [40]. Based on WHtR as reference, pBMI was less sensitive (54.0%) to identifying obesity but was highly specific (97.5%) in discriminating non-obese adolescents in the population. This finding corroborates previous studies that have shown low sensitivity of pBMI in screening obesity in Ghanaian children when percentage of body fat was used as reference [41].

ROC analysis for assessing anthropometric measures showed WC as the best indicator for obesity, followed by pBMI and HC, when WHtR was used as reference. As such, the optimal cut-off values for identifying obesity by NC, MUAC, WC, HC and pBMI in the cohort were 30.6 cm, 25.9 cm, 75.1 cm, 92.15 cm and p85.2th, respectively. On the other hand, WHtR was the best indicator for obesity, followed by WC and HC, when pBMI was used as reference. Thus, the optimal cut-offs for obesity by WHtR, NC, MUAC, WC and HC in the cohort were 0.481, 30.95 cm, 27.95 cm, 76.1 cm and 95.75 cm, respectively, in the cohort. The observed cut-off score for WHtR (0.481) in the cohort was slightly different but close to the recommended standard of 0.5, particularly in males (0.49), but also females (0.484). This suggests that the recommended cut-off score of 0.5 for WHtR may not be affected by ethnicity in screening obesity. This finding supports previous studies, which have shown that WHtR is less affected by sex or ethnicity [42], as similar cut-off values have been observed in different ethnic populations [43,44]. Moreover, a previous study by Matsha et al. in 2013 [45] in South African children aged 10–16 years showed a WHtR cut-off of 0.465, lower than the recommended 0.5 cut-off and the scores of this study (0.481). The steady increase of the prevalence of obesity in South African children and adolescents reported may have resulted in a higher cut-off value for WHtR. Therefore, routine assessments of obesity indicators at different time points may be necessary.

Furthermore, a lower cut-off value of p85.2th (p84.45th for females and p80.75th for males) with improved sensitivity (87.9%) was observed for pBMI. This value is considerably different from the CDC recommended cut-off score of p95th for obesity in children and adolescents. A previous study equally showed a lower cut-off for pBMI in Chinese children [46], which was also different from the CDC cut-off value. The present study suggests that the CDC recommended BMI percentile (p95th) cut-off value for the diagnosis of (or classifying) obesity in children may not account for gender, ethnic and genetic differences. BMI percentile has been suggested to be affected by body composition in children, as the distribution of fat mass and fat-free mass is highly variable and affected by age and pubertal maturation, wherein adiposity may be linked with advanced puberty in girls [47]. Postnatal linear growth pattern for weight and height is controlled by genetic, nutrition and endocrine factors that may influence the onset of puberty [48]. A study in South Africa, which assessed body composition and abdominal adiposity from birth to age 22, showed that relative linear growth was associated with fat mass as well as abdominal, visceral and subcutaneous adipose tissues in children 0–8 years and 19–22 years but showed no association in the adolescent age group (8–18 years) [34]. This implies that at adolescence there is variability of body composition, confirming the influence of puberty on the linear growth pattern. Moreover, epidemiological reports have suggested that the timing of pubertal onset may be a key determinant of cardio-metabolic health in adolescence and adulthood, and there may exist racial and ethnic disparities of puberty onset timing [49]. Considering that puberty onset commences at adolescence, the disparity of pBMI cut-off obtained in this study to that of CDC cut-off may be accounted for by puberty, genetic and ethnic differences.

Among the other anthropometric measures (WC, NC, MUAC and HC), WC showed a high sensitivity and specificity and could be an alternative and feasible method to screen obesity in adolescents, applying a proposed cut-off range of between 75.1 and 76.1 cm, which was higher in females than in males. Previous studies have suggested WC to be a good indicator for obesity screening in children [50,51]. However, there is no recommended cut-off score for WC in the assessment of obesity in children. Therefore, the suggested cut-off value may be used in conjunction with WHtR and/or pBMI indicators. In an Iranian study, NC was shown to be a useful indicator in screening obesity in children and adolescents, with cut-off values of 27.5–38.3 cm for males and 26.7–33.4 cm for females [52]. In the present study, NC was shown to have the lowest sensitivity and thus was a poor indicator for screening obesity in adolescents and showed cut-off values ranging between 30.6 and 31.85 cm, slightly higher in males. The cut-off scores, however, fall within the range of other previous studies in children, as reported by Teheri et al. [52] MUAC has been reported as a possible tool for screening obesity [53] in children. In the present study, MUAC was not a good indicator for obesity. The cut-off observed in this study ranged from 25.55 to 27.75 cm. This finding concords with a 12-country study reporting an approximately 25 cm cut-off value for MUAC in both boys and girls aged 9–11 years and was country-specific, with South African boys presenting a cut-off of 23.2 cm [54]. A lower cut-off for MUAC between 16.38 and 22.73 cm was observed in Pakistani children aged 5–14 years [55]. Moreover, MUAC has been shown to be appropriate in screening overweight status in children younger than 2 years of age in a South African population with a high sensitivity and specificity [56]. However, the sensitivity of MUAC in the present study was relatively low and thus not a good indicator to screen obesity in this population. HC has rarely been used as an indicator for obesity, as it has mostly been used to determine waist-to-hip ratio (WHR), which is a good indicator for obesity in adults. We established cut-off values ranging from 92.15 to 96.75 cm, though HC showed a low sensitivity compared to pBMI, WHtR and WC. Interestingly, its sensitivity was higher than that of MUAC and NC. This result is therefore indicative of a possible usefulness of HC to screen obesity in children. However, this will require several studies to assess HC for obesity screening in order to develop recommended cut-off values in male and female children.

With regards to age-wise percentile distribution for the anthropometric measures as presented in Table 8, percentiles increased with age and differed between male and female children. The 95th percentile of all the anthropometric indices presented values that were above the established cut-offs for the various anthropometric indicators. Moreover, it was in the 85th percentile for WHtR that values were comparable to the 0.5 cut-off for most age groups in both males and females. For pBMI, the 95th percentile showed values above the p95th cut-off for all age groups, while it was the 85th percentile that showed values similar to the p95th cut-off for some age groups. This finding suggests that values at the 85th percentile may be a suitable indicator for obesity for the various anthropometric indices.

This is the first study to assess obesity indicators for South African adolescents of African ancestry. This study has shown that the established pBMI cut-off in this population was different from the CDC recommended cut-off. Thus, the CDC cut-off value pBMI may be affected by race and ethnic differences in adolescents. However, the findings of this study may be limited, in that it did not use a reference method for the direct quantification of body fat but relied on WHtR as a reference method, which depends on central adiposity for assessing obesity in children and has been shown to be a better indicator than pBMI. Moreover, our study did not assess other cardiometabolic disease risk factors, such as dyslipidaemia, hypertension and insulin resistance, which are associated with obesity and may associate differently with anthropometric measures in different populations. More so, this study constituted South African adolescents of African ancestry who are predominately of the Xhosa ethnicity and therefore did not include other ethnic populations in South Africa. Hence, the findings may be limited to a generalization of the entire South African adolescent population.

## 5. Conclusions

This study reveals a lower pBMI cut-off value, different from the CDC recommended cut-off, for screening obesity in a South African adolescent population. This study has established that the optimal pBMI cut-off for obesity screening may be ethnic-specific. This study was limited to South Africans of African ancestry predominately of the Xhosa ethnic group. Therefore, future studies involving other ethnic populations with larger sample sizes are recommended to provide national representative data for the South African adolescent population.

## Figures and Tables

**Table 1:** Baseline characteristics of the study population.

	Females (*n* = 796)			Males (*n* = 348)			
	Mean ± SD	CI	Min.-Max.	Mean ± SD	CI	Min.-Max.	*p*-Value
**Age (years)**	13.88 ± 1.61	13.77, 14.00	11–17	13.17 ± 1.70	12.99, 13.35	11–17	0.001
**Weight (kg)**	55.38 ± 15.11	54.32, 56.43	26.7–112.50	50.97 ± 15.32	49.36, 52.59	24.0–108.69	0.079
**Height (m)**	1.56 ± 0.08	1.56, 1.57	1.31–1.88	1.56 ± 0.11	1.55, 1.57	1.28–1.86	0.003
**BMI (m/h^2^)**	22.43 ± 5.30	22.06, 22.80	13.55–42.00	20.57 ± 4.73	20.07, 21.07	13.50–41.50	0.001
**pBMI**	64.83 ± 28.87	62.81, 66.85	0.6–99.8	56.79 ± 31.17	53.50, 60.09	0.1, 99.6	0.001
**WHtR**	0.47 ± 0.07	0.46, 0.47	0.33–0.77	0.45 ± 0.06	0.45, 0.46	0.35–0.71	0.010
**WC (cm)**	72.94 ± 11.10	72.17, 73.71	50.0–119.0	70.64 ± 10.77	69.50, 71.77	52.0–115.5	0.117
**HC (cm)**	92.05 ± 13.23	91.13, 92.97	53.0–136.5	85.05 ± 11.87	85.79, 88.30	49.4–128.0	0.001
**Neck (cm)**	29.9 ± 2.32	29.70, 30.11	23.5–42.5	29.97 ± 2.57	29.67, 30.27	22.5–38.0	0.342
**MUAC (cm)**	24.54 ± 4.44	24.14, 24.94	12.5–43.5	23.14 ± 4.89	22.58, 23.17	13.0–35.7	0.001

CI: confidence interval, HC: hip circumference, MUAC: mid-upper arm circumference, NC: neck circumference, WC: waist circumference, WHtR: waist-to-height ratio, pBMI: body mass index percentile.

**Table 2:** Prevalence of obesity based on WHtR and BMI percentiles.

*n* (%)	Category	Cohort 1144 (%)	Female 796 (%)	Male 348 (%)
**WHtR**	Non-obese	855 (74.7)	579 (72.7)	276 (79.3)
	Obese	289 (25.3)	217 (27.3)	72 (20.7)
**pBMI**	Non-obese	967 (84.5)	663 (83.3)	308 (87.4)
	Obese	177 (15.5)	133 (16.7)	44 (12.6)

WHtR: waist-to-height ratio, pBMI: body mass index percentile.

**Table 3:** Body weight classification by BMI percentile split by age and sex.

*n* (%)	Underweight	Normal Weight	Overweight	Obese	Total
**Sex**					
**Female**	17 (2.1)	508 (63.8)	138 (17.3)	133 (16.7)	796
**Male**	20 (5.7)	236 (67.8)	48 (13.8)	44 (12.6)	348
**Cohort (total)**	37 (3.2)	744 (65.0)	186 (16.3)	177 (15.5)	1144
**Age**					
**11**	6 (5.9)	59 (58.4)	21 (20.8)	15 (14.9)	101
**12**	12 (5.6)	155 (72.4)	24 (11.2)	23 (10.7)	214
**13**	9 (3.2)	193 (68.9)	38 (13.6)	40 (14.3)	280
**14**	4 (2.2)	119 (65.7)	27 (14.9)	31 (17.1)	181
**15**	5 (3.0)	98 (59.0)	39 (23.5)	24 (14.5)	166
**16**	1 (0.7)	83 (58.5)	23 (16.2)	35 (24.6)	142
**17**	0 (0.0)	37 (61.7)	14 (23.3)	9 (15.0)	60
**Cohort (total)**	37 (3.2)	744 (65.0)	186 (16.3)	177 (15.5)	1144

**Table 4:** Sensitivity and specificity of BMI percentile in screening obesity using WHtR as reference.

				WHtR			
		Cohort		Female		Male	
		**Obese (%)**	**Non-obese (%)**	**Obese (%)**	**Non-obese (%)**	**Obese (%)**	**Non-obese (%)**
	**Obese**	156 (54.0)	21 (2.5)	115 (53.0)	18 (3.1)	41 (56.9)	3 (1.1)
**pBMI**	**Non-obese**	133 (46.0)	834 (97.5)	102 (47.0)	561 (96.9)	31 (43.1)	273 (98.9)
	**Total**	289	855	217	579	72	276
		**Cohort**		**Female**		**Male**	
		**Sensitivity%**	**Specificity%**	**Sensitivity%**	**Specificity%**	**Sensitivity%**	**Specificity%**
**pBMI**		54.0	97.5	53.0	96.9	56.9	98.9

WHtR: waist-to-height ratio, pBMI: body mass index percentile.

**Table 5:** Optimal cut-off, sensitivity, specificity, SE and area under the ROC curves for anthropometric indices in predicting obesity in males, females and cohort using WHtR.

	Cut-off	Sensitivity%	Specificity%	AUC	SE	95%CI
**Cohort**						
**NC**	30.6	71.3	77.6	0.786	0.021	0.745–0.827
**MUAC**	25.9	80.3	79.8	0.813	0.026	0.762–0.865
**WC**	75.1	90.4	89.4	0.982	0.004	0.974–0.991
**HC**	92.15	80.3	84.7	0.883	0.018	0.848–0.917
**pBMI**	85.2	87.9	88.9	0.947	0.010	0.927–0.967
**pBMIr**	95.1	57.3	98.3	0.947	0.010	0.927–0.967
**Female**						
**NC**	30.70	72.2	77.9	0.801	0.025	0.752–0.851
**MUAC**	25.55	85.6	76.3	0.853	0.028	0.798–0.908
**WC**	76.1	91.8	95.4	0.982	0.006	0.970–0.994
**HC**	90.25	85.6	75.7	0.864	0.024	0.817–0.912
**pBMI**	84.45	90.7	87.2	0.947	0.013	0.922–0.972
**pBMIr**	95.1	56.7	98.1	0.947	0.013	0.922–0.972
**Male**						
**NC**	30.6	70.0	85.4	0.764	0.037	0.691–0.837
**MUAC**	25.90	71.7	85.0	0.749	0.050	0.650–0.847
**WC**	73.25	95.0	91.2	0.985	0.006	0.973–0.996
**HC**	93.50	78.3	93.0	0.915	0.025	0.867–0.963
**pBMI**	80.75	90.0	87.2	0.946	0.017	0.913–0.980
**pBMIr**	95.0	58.3	98.7	0.946	0.017	0.913–0.980

AUC: area under the curve, SE: standard error, CI: confidence interval, HC: hip circumference, MUAC: mid-upper arm circumference, NC: neck circumference, WC: waist circumference, WHtR: waist-to-height ratio, pBMI: body mass index percentile, pBMIr: recommended body mass index percentile.

**Table 6:** Optimal cut-off, sensitivity, specificity, SE and area under the ROC curves for anthropometric indices in predicting obesity in males, females and cohort using pBMI.

	Cut-off	Sensitivity%	Specificity%	AUC	SE	95%CI
**Cohort**						
**WHtR**	0.481	97.0	84.0	0.966	0.007	0.952–0.980
**NC**	30.95	82.2	73.4	0.837	0.023	0.793–0.882
**MUAC**	27.95	85.1	90.8	0.909	0.023	0.864–0.955
**WC**	76.1	91.1	89.3	0.965	0.008	0.949–0.980
**HC**	95.75	90.1	87.1	0.934	0.017	0.900–0.968
**Female**						
**WHtR**	0.484	96.8	84.1	0.965	0.008	0.949–0.981
**NC**	30.95	84.1	95.6	0.846	0.026	0.795–0.897
**MUAC**	27.95	93.7	89.8	0.851	0.017	0.920–0.985
**WC**	76.1	92.1	87.6	0.966	0.008	0.949–0.982
**HC**	95.25	90.5	83.4	0.922	0.023	0.877–0.967
**Male**						
**WHtR**	0.491	97.4	88.8	0.968	0.014	0.940–0.995
**NC**	31.85	71.1	82.5	0.827	0.042	0.745–0.909
**MUAC**	25.9	84.2	82.1	0.842	0.052	0.740–0.944
**WC**	75.75	94.7	90.0	0.963	0.016	0.932–0.995
**HC**	96.75	89.5	93.2	0.954	0.026	0.902–1.006

AUC: area under the curve, SE: standard error, CI: confidence interval, HC: hip circumference, MUAC: mid-upper arm circumference, NC: neck circumference, WC: waist circumference, WHtR: waist-to-height ratio, pBMI: body mass index percentile.

**Table 7:** Predicted prevalence of obesity based on the new cut-off values using WHtR and pBMI.

	Cohort (%)	Female (%)	Male (%)
**Ref: WHtR**			
**NC**	263 (23.0)	155 (19.5)	108 (31.0)
**MUAC**	249 (21.8)	177 (22.3)	77 (22.1)
**WC**	357 (31.2)	250 (31.4)	107 (30.7)
**HC**	438 (38.3)	296 (37.2)	89 (25.6)
**BMI%**	352 (30.8)	274 (34.4)	105 (30.2)
**Ref: pBMI**			
**NC**	260 (22.7)	153 (19.2)	72 (20.7)
**MUAC**	147 (12.9)	101 (12.7)	77 (22.1)
**WC**	323 (28.2)	250 (31.4)	81 (23.3)
**HC**	367 (32.1)	290 (36.4)	73 (21.0)
**WHtR**	342 (29.9)	259 (32.5)	73 (21.0)

Ref: reference, HC: hip circumference, MUAC: mid-upper arm circumference, NC: neck circumference, WC: waist circumference, WHtR: waist-to-height ratio, pBMI: body mass index percentile.

**Table 8:** Percentiles of the various obesity measures in children.

			FEMALE			MALE		
	WHtR	pBMI	WC	HC	WHtR	pBMI	WC	HC
**11 yrs**								
**5th**	0.386	4.80	61.00	70.15	0.382	3.80	56.00	65.00
**25th**	0.421	27.40	64.75	77.75	0.413	35.60	61.00	76.00
**50th**	0.452	70.20	69.00	81.00	0.445	69.50	68.00	85.00
**75th**	0.494	90.50	74.50	95.00	0.507	91.80	76.00	91.00
**85th**	0.519	94.70	78.55	98.20	0.542	95.40	80.00	97.00
**95th**	0.571	97.90	90.25	106.70	0.636	98.60	98.00	109.00
**12 yrs**								
**5th**	0.376	5.30	57.00	72.00	0.378	1.40	57.00	70.00
**25th**	0.410	27.80	62.00	75.88	0.413	34.10	62.00	76.25
**50th**	0.435	53.20	66.00	84.00	0.437	54.00	65.00	83.50
**75th**	0.482	82.90	73.25	91.00	0.478	81.90	72.00	91.75
**85th**	0.521	93.22	81.00	97.35	0.506	93.14	78.00	98.25
**95th**	0.610	98.18	97.45	110.25	0.596	98.68	92.50	105.75
**13 yrs**								
**5th**	0.377	6.56	56.90	70.75	0.382	8.71	58.00	70.24
**25th**	0.412	39.55	63.00	78.38	0.409	27.90	62.00	78.00
**50th**	0.448	68.40	68.00	85.00	0.439	61.80	67.00	83.00
**75th**	0.485	90.15	75.00	96.13	0.471	87.10	74.25	92.25
**85th**	0.531	95.00	81.60	101.88	0.518	92.11	81.00	98.10
**95th**	0.583	97.83	89.60	108.25	0.605	98.94	92.70	111.70
**14 yrs**								
**5th**	0.368	8.49	58.05	75.15	0.382	10.45	60.20	69.60
**25th**	0.413	47.25	65.00	85.00	0.409	34.00	65.25	81.00
**50th**	0.451	69.05	71.00	90.25	0.433	62.20	70.00	84.00
**75th**	0.505	92.45	79.25	99.00	0.501	90.15	74.50	93.50
**85th**	0.528	95.00	84.00	104.00	0.522	95.21	83.75	101.49
**95th**	0.595	97.59	90.98	112.00	0.596	99.07	94.95	115.95
**15 yrs**								
**5th**	0.379	14.93	60.90	79.00	0.357	0.25	57.75	67.00
**25th**	0.421	52.25	67.00	88.00	0.379	19.10	62.50	80.00
**50th**	0.460	79.05	74.00	95.00	0.422	54.40	70.00	85.00
**75th**	0.505	91.68	81.50	104.25	0.461	78.75	75.85	94.35
**85th**	0.543	95.00	85.54	109.60	0.532	91.85	84.00	100.50
**95th**	0.596	95.41	97.70	119.10	0.581	98.10	97.25	112.65
**16 yrs**								
**5th**	0.388	11.73	63.25	83.00	0.381	5.50	64.47	79.93
**25th**	0.433	62.25	71.13	91.00	0.403	30.30	67.75	84.00
**50th**	0.481	83.00	76.70	100.75	0.421	63.00	72.00	89.00
**75th**	0.537	95.00	84.00	111.90	0.473	85.50	80.00	98.50
**85th**	0.563	95.00	92.00	116.00	0.515	93.60	87.38	105.38
**95th**	0.609	96.98	97.38	124.85	0.599	97.76	102.68	115.35
**17 yrs**								
**5th**	0.396	14.30	65.45	85.45	0.379	5.00	67.00	81.00
**25th**	0.443	62.50	71.63	90.25	0.412	26.00	69.25	88.15
**50th**	0.475	81.50	76.00	99.50	0.425	57.50	73.75	91.75
**75th**	0.526	92.75	84.75	106.00	0.477	79.75	83.75	102.75
**85th**	0.562	95.00	87.33	109.83	0.514	93.05	89.15	108.70
**95th**	0.608	95.00	93.10	122.37	0.519	94.00	92.00	112.50

HC: hip circumference, WC: waist circumference, WHtR: waist-to-height ratio, pBMI: body mass index percentile.

## Data Availability

All the data for this study have been summarised in the results. According to the South Africa National Data Protection Act, all of the participant’s data are kept confidential and may be available upon reasonable request.
